# Bronchial thermoplasty reduces ventilation heterogeneity measured by phase-resolved functional lung magnetic resonance imaging in severe asthma

**DOI:** 10.1186/s12931-025-03372-w

**Published:** 2025-10-21

**Authors:** Chuan T. Foo, David Langton, Graham M. Donovan, Bruce R. Thompson, Peter B. Noble, Francis Thien

**Affiliations:** 1 Faculty of Medicine, Nursing and Health Sciences, Monash University, Melbourne, VIC Australia; 2 Department of Respiratory Medicine, Eastern Health, Melbourne, VIC Australia; 3 Department of Thoracic Medicine, Peninsula Health, Frankston, VIC Australia; 4 Department of Mathematics, University of Auckland, Auckland, New Zealand; 5 School of Health Sciences, University of Melbourne, Melbourne, VIC Australia; 6 School of Human Sciences, The University of Western Australia, Perth, WA Australia

**Keywords:** Asthma, Bronchial thermoplasty, Functional lung imaging, Pathophysiology, PREFUL, Ventilation heterogeneity

## Abstract

**Rationale:**

Bronchial thermoplasty (BT) is a treatment option for patients with severe asthma. Despite demonstrated sustained symptomatic benefits, its mechanism of action remains unclear, with emerging evidence suggesting a reduction in ventilation heterogeneity.

**Objective:**

This study aims to determine if BT reduces ventilation heterogeneity as measured by phase-resolved function lung magnetic resonance imaging (PREFUL MRI).

**Methods:**

Twenty-one patients with severe asthma and 14 healthy volunteers (HV) were recruited. Patients were assessed at baseline and 6-months after BT. Data collected included asthma control questionnaire (ACQ-5), exacerbation frequency, and short-acting beta-agonist (SABA) and oral corticosteroid (OCS) use. Both HV and patients also underwent lung function tests and PREFUL MRI. Ventilation heterogeneity was assessed using ventilation defect percentage (VDP) of static regional ventilation (RVent) and dynamic flow-volume loop cross-correlation metric (FVL-CM), and interquartile distance (IQD) of the ventilation distribution.

**Results:**

At baseline, patients had a significantly higher RVent VDP (20.0 ± 14.5 vs 3.8 ± 2.2%, *p* < 0.001), FVL-CM VDP (23.7 ± 17.8 vs 2.4 ± 2.3%, *p* < 0.001), and IQD (0.61 ± 0.27 vs 0.32 ± 0.05, *p* < 0.001) than HV. Post BT, significant reductions in RVent VDP (15.5 ± 11.7 vs 20.0 ± 14.5%, *p* < 0.001), FVL-CM VDP (18.7 ± 13.9 vs 23.7 ± 17.8%, *p* < 0.001), and IQD (0.53 ± 0.22 vs 0.61 ± 0.27, *p* < 0.001) were observed in patients, along with significant improvements in ACQ-5, exacerbation frequency, SABA and OCS use. No change in lung function was seen. Significant correlations were observed between ΔACQ and ΔRVent VDP (ρ = 0.50, *p* = 0.02), ΔFVL-CM VDP (ρ = 0.51, *p* = 0.02), and ΔIQD (ρ = 0.45, *p* = 0.04).

**Conclusions:**

Clinical benefits post BT are accompanied by a reduction in ventilation heterogeneity that are undetected by lung function test. These findings provide valuable insights into the mechanisms of action of BT and highlight the complementary role of functional lung imaging in the study of pulmonary diseases for which traditional lung function tests may be insensitive at detecting therapeutic response.

## Introduction

Bronchial thermoplasty (BT) is a bronchoscopic intervention for the treatment of severe asthma, and involves the direct application of radiofrequency energy to the airway mucosa, resulting in thermal ablation and shrinking of the underlying airway smooth muscle (ASM) [1–3]. BT has been shown to reduce asthma exacerbations and improve asthma symptoms and quality of life, with benefits sustained for up to 10 years [1, 4–8]. Despite producing sustained improvement in asthma control, there is great uncertainty surrounding BT’s mechanism of action, especially given only a fraction of the total airways are treated, with spirometry showing little or no change post treatment [1, 6, 9–11].

Emerging evidence from modelling and physiological studies suggest that BT reduces ventilation heterogeneity [12, 13], thereby improving gas exchange. The theory behind this hypothesis is that various compartments of the lung are interdependent, allowing pathologies in one region to affect another. In the context of BT, dilation of the proximal airways treated during BT should lead to a reopening cascade in the smaller distal untreated airways, resulting in a redistribution of airflow that reduces ventilation heterogeneity [13]. Indeed, imaging studies have observed dilation of proximal airways [14–17] and reductions in regions of low or absent ventilation in patients after BT [18, 19]. Reductions in multiple breath nitrogen washout and gas trapping have also been reported [20, 21]. Finally, an exploratory study combining computed tomography (CT) functional respiratory imaging with a modified BT protocol described reductions in ventilation heterogeneity after treatment of the left, but not the untreated right lung [12]. Whilst these are promising developments, imaging studies demonstrating a reduction in ventilation heterogeneity post BT are still lacking.

A number of functional lung imaging techniques are currently available to evaluate lung ventilation heterogeneity. Of these, hyperpolarized gas magnetic resonance imaging (MRI) is the most established and considered the current gold standard, although its use is limited to research institutions due to its high upfront cost, complex imaging pipeline and need for skilled personnel [22]. Phase-resolved functional lung (PREFUL) MRI is a relatively new imaging modality that utilizes the changes in endogenous lung MR signal during tidal respiration as a proxy for lung ventilation [23]. Compared to hyperpolarized gas MRI, the advantages of PREFUL MRI are its low cost (PREFUL MRI can be performed on any standard clinical MRI scanner), short scanning time, and the ability to perform in patients without the requirement for breath-holding maneuvers [22]. PREFUL MRI has been studied in a variety of patient populations – asthma [24, 25], chronic obstructive pulmonary disease [26–29], cystic fibrosis [30–33], chronic lung allograft dysfunction [34, 35] and COVID-19 [36, 37] – and has been shown to be sensitive to treatment [24–27, 31, 33, 38], and correlate well with lung function tests [24, 25, 27, 28, 30–33] and other imaging modalities including hyperpolarized ^129^Xe MRI [25, 28, 30, 32, 33]. Routinely derived functional parameters include regional ventilation (RVent), flow volume loop cross-correlation metric (FVL-CM) and their respective ventilation defect percentages (VDP) [29, 39], with inhomogeneity index and interquartile distance of the ventilation distribution (IQD) more recently reported [24].

The objective of the current study is to examine the hypothesis that BT, as an accepted therapy reducing ASM remodelling in large airways, reduces ventilation heterogeneity assessed by PREFUL MRI. Successful detection of changes in ventilation heterogeneity using PREFUL MRI would also contribute to the growing body of evidence on the utility of PREFUL MRI in patient selection and assessing response to therapy in asthma and other lung diseases.

## Methods

### Participants and study design

This prospective study enrolled patients with severe asthma and healthy volunteers at two Australian tertiary hospitals. Assessments were conducted at baseline (pre-BT) and 6-months post BT for patients with asthma, and only at baseline for healthy volunteers. At each assessment, participants completed a standardized questionnaire and lung function tests on the first visit, and PREFUL MRI on the second visit. The two visits were conducted no longer than five days apart. The study was approved by the Eastern Health Institutional Review Board (E21-021–79977) and conducted in accordance with The Code of Ethics of the World Medical Association (Declaration of Helsinki). All participants were aged 18 years and above and provided written informed consent.

Patients had to have a diagnosis of severe asthma as defined by the European Respiratory Society/American Thoracic Society (ERS/ATS), and be scheduled to undergo BT as part of their clinical care [40]. Specifically, all patients selected for BT needed to show evidence of uncontrolled asthma such as high symptom burden (5-item Asthma Control Questionnaire (ACQ) ≥ 2) or frequent asthma exacerbations (≥ 2 oral corticosteroid (OCS) requiring episodes) in the preceding 12 months despite receiving Global Initiative for Asthma (GINA) Step 4–5 treatment for at least 12 months [41]. Healthy volunteers had to be never smokers with no history of any health conditions.

Exclusion criteria included participants who were (i) < 18 years old; (ii) females who were pregnant or lactating; (iii) unable to provide written informed consent; (iv) unable or unwilling to undergo MRI; and (v) had an alternative respiratory condition such as interstitial lung disease, chronic obstructive pulmonary disease, or bronchiectasis.

### Bronchial thermoplasty procedure

BT was performed in three separate procedures, scheduled 3–4 weeks apart using the Alair Bronchial Thermoplasty System (Boston Scientific Corporation (BSC), USA) under deep sedation or general anaesthesia by an experienced bronchoscopist [1]. All patients received 50 mg oral prednisolone three days prior to the procedure and were routinely admitted to hospital for observation post-procedure.

### Clinical measurements and lung function tests

Data collected included demographics, asthma medication usage, asthma exacerbation frequency, 5-item ACQ, and lung function parameters. Data were collected at baseline and 6 months after completion of BT.

Pre- and post-bronchodilator spirometry were performed in all participants, with patients withholding their usual bronchodilators for 12–24 h prior to testing. Diffusing capacity of the lung for carbon dioxide (DLCO) was also measured in patients. All tests were performed in accordance with ERS/ATS standards [42]. Predicted value equations were taken from the Global Lung Initiative [43–45].

### PREFUL MRI

Image acquisition was performed on a 3 T Siemens Magnetom Skyra (Siemens Healthineers, Erlangen, Germany) with an 18-channel body array coil. A spoiled gradient echo sequence was used with the following scan parameters: field of view 500 × 500 mm2, matrix size 128 × 128, slice thickness 15 mm, echo time 0.83 ms, repetition time 2.1 ms, bandwidth 1860 Hz/pixel and flip angle 4°. Five coronal slices were acquired, including one centred on the carina, two anterior to the carina, and two posterior to the carina. All slices were contiguous with no gap. Each slice comprised of 250 frames acquired during tidal respiration. The total scan duration was 175 s at a temporal resolution of 135 ms/frame. All participants were imaged pre-bronchodilator, with patients withholding their regular bronchodilators for 12–24 h prior to imaging.

MRI images were analysed using a graphical user interface application based on the PREFUL analysis pipeline described by Voskrebenzev et al. [23, 46]. The application performs the necessary image registration, segmentation, filtering, phase sorting and biomarker extraction. Lung ventilation was quantified using RVent and FVL-CM. VDP was calculated as the percent of the lung/voxels below 40% of the 90th percentile of the cumulative MR signal intensity distribution for RVent, and below a fixed threshold of 0.9 for FVL-CM [29, 39].

Additionally, masked PREFUL ventilation images were processed in MATLAB (R2023b, MathWorks, Natick, Massachusetts, USA) to generate voxel-value histograms after excluding extreme outliers (values above the 99th percentile). The differences in the shape and spread of the histogram offers insights into the changes in ventilation distribution before and after BT, and between participant groups. Other heterogeneity parameters including IQD (defined as the inter-quartile range divided by the mean) was also calculated. Collectively, RVent VDP, FVL-CM VDP, and IQD provide information on ventilation heterogeneity.

### Statistical analysis

Continuous data was summarised using mean, median, standard deviation or interquartile range depending on the normality of their distributions, assessed by the Shapiro–Wilk test. Categorical data were presented as number and percentage. Between group comparisons were performed using Student’s *t-*test or Mann Whitney *U*-test for continuous variables and χ^2^ or Fisher’s exact test for categorical variables. Paired group measurements were tested for significance using paired *t*-test or Wilcoxon signed-rank test. Correlation analyses were conducted using either Pearson or Spearman correlation. The Hochberg method was used to adjust for multiple comparisons. All statistical analyses were performed using SPSS Version 29 (IBM corporation, New York, USA). A *P* value of < 0.05 was considered statistically significant.

## Results

### Baseline demographics and characteristics

Twenty-five patients with severe asthma and 14 healthy volunteers were recruited for this study. Four patients were excluded due to the following reasons: (i) two patients did not proceed with BT after completing baseline assessments; (ii) one patient experienced a massive pulmonary embolism after the first BT session and did not proceed with further BT; and (iii) one patient died from brain malignancy before completing follow-up. Twenty-one patients (17 females, 4 males) and 14 healthy volunteers (10 females, 4 males) completed research assessments and were included in the final analysis.

Baseline characteristics and medication use are summarised in Tables 1 and 2. Patients were significantly older (50.8 ± 17.8 vs 29.9 ± 11.2 years, *P* < 0.001), and had higher BMI (31.9 ± 7.2 vs 23.0 ± 2.6 kg/m^2^, *P* < 0.001) than healthy volunteers. None of the patients were active smokers – 13 never smokers; 7 with a < 5 packet year history; and 1 with a > 5 packet year history. All healthy volunteers were never smokers by definition. No significant sex difference was observed between groups.

**Table 1:** Baseline characteristics

	Patients (*N* = 21)	Healthy Volunteers (*N* = 14)	*P* Value
Age (years)	50.8 ± 18.8	29.9 ± 11.2	< 0.001
Males/Females	4 (19)/17 (81)	4 (28.6)/10 (71.4)	0.51
BMI (kg/m^2^)	31.9 ± 7.2	23.0 ± 2.6	< 0.001
Smoking status			
Never smoker	13 (61.9)	14 (100)	0.009
Ex-smoker	8 (38.1)		
Beclomethasone-equivalent dose (ug/day)	1961.9 ± 873.2	-	
Maintenance OCS	5 (23.8)	-	
Asthma biologic			
At time of 1 st BT session	7 (33.3)	-	
Previously discontinued	6 (28.6)	-	

Values are presented as mean ± standard deviation or number (%)

*BT* bronchial thermoplasty, *BMI* body mass index; OCS, oral corticosteroid

**Table 2:** Key clinical outcomes pre and post BT

**Clinical variable**	Healthy volunteers (*N* = 14)	Severe asthma patients (*N* = 21)		*P* Value	
	Baseline	Baseline	6-months	HV vs SA	SA*
ACQ	-	2.8 (2.2–3.4)	1.4 (1.0–2.0)	-	< 0.001
SABA usage (puffs/day)	-	10 (3–16)	2 (0.4–10)	-	0.001
Maintenance OCS (mg/day)	-	10 (7.5–50)	5 (2.5–17.5)	-	-
OCS-requiring exacerbations	-	2.5 (1–4.5)	1 (0–2)	-	< 0.001
Lung function variable					
Pre-bronchodilator FEV1 z-score/ % predicted	−0.07 ± 1.13/ 99.1 ± 13.5	−1.95 ± 1.61/ 70.6 ± 24.8	−1.99 ± 1.41/ 71.2 ± 21.8	< 0.001/ < 0.001	0.79/ 0.82
Pre-bronchodilator FVC z-score/ % predicted	0.11 ± 1.30/ 101.7 ± 15.3	−0.99 ± 1.29/ 86.4 ± 17.2	−1.04 ± 1.06/ 87.7 ± 15.4	0.02/ 0.01	0.67/ 0.45
Pre-bronchodilator FER z-score/ FER%	−0.33 ± 1.10/ 83.6 ± 7.3	−1.95 ± 1.58/ 64.4 ± 15.0	−1.82 ± 1.47/ 64.3 ± 15.2	0.002/ < 0.001	0.68/ 0.99
Bronchodilator response in FEV1 (%)	3.6 ± 2.4	19.7 ± 36.0	8.21 ± 15.6	0.06	0.15
KCO z-score/ % predicted	-	0.65 ± 1.64/ 110.4 ± 23.5	0.37 ± 1.51/ 106.1 ± 19.9	-	0.08/ 0.09
Ventilation variable					
RVent VDP (%)	3.8 ± 2.2	20.0 ± 14.5	15.5 ± 11.7	< 0.001	0.04
FVL-CM VDP (%)	2.4 ± 2.3	23.7 ± 17.8	18.7 ± 13.9	< 0.001	0.04
IQD	0.32 ± 0.05	0.61 ± 0.27	0.53 ± 0.22	< 0.001	0.04

*ACQ* Asthma control questionnaire, *BT* Bronchial thermoplasty, *FEV1* Forced expiratory volume in 1 s, *FER* Forced expiratory ratio, *FVC* Forced vital capacity, *FVL-CM* Flow volume loop cross-correlation metric, *HV* Healthy volunteers, *IQD* Interquartile distance, *KCO*, mean diffusion capacity of the lung for carbon monoxide per unit lung volume, *OCS* Oral corticosteroids, *RVent* Regional ventilation, *SABA* Short-acting beta-agonist, *SA* Severe asthma patients, *VDP* Ventilation defect percentage

^*^Comparing baseline with 6-months post BT values

Patients had a high symptom burden with a median ACQ of 2.8 (2.2–3.4) despite using triple inhaler therapy with beclomethasone-equivalent ICS dose 1962 ± 873 ug/day. Seven (33.3%) were receiving treatment with an asthma biologic at the time of the 1 st BT session and 5 (23.8%) were taking maintenance OCS with a group median dose of 10 (7.5–50) mg/day. The median daily requirement for short-acting beta-agonist (SABA) therapy was 10 (3–16) puffs/day. The frequency of OCS requiring exacerbations in the 6 months prior to BT was 2.5 (1–4.5).

At baseline, patients had a mean pre-bronchodilator forced expiratory volume in 1 s (FEV1) z-score of −1.95 ± 1.61 (70.6 ± 24.8% predicted), with an average bronchodilator response of 19.7 ± 36.0%. Vital capacity z-score was −0.99 ± 1.29 (86.4 ± 17.2% predicted) and forced expiratory ratio z-score was −1.95 ± 1.58 (64.4 ± 15.0%). The mean DLCO per unit lung volume z-score was 0.65 ± 1.64 (110.4 ± 23.5% predicted). Compared to healthy volunteers, patients had significantly lower lung function (Table 2).

### Post treatment outcomes

All BT procedures were well tolerated. Apart from one patient who experienced a massive pulmonary embolism after the first BT session, there were no other unexpected complications. An average of 298 ± 83 radio-frequency activations were delivered per patient.

Significant improvements in ACQ, maintenance OCS dose, number of OCS-requiring exacerbations, and SABA usage were noted 6-months after completion of BT compared to baseline (Table 2). Median ACQ was reduced from 2.8 (2.2–3.4) to 1.4 (1.0–2.0) (*P* < 0.001), and three out of five (60%) patients who required maintenance OCS at baseline were able to reduce their OCS dose, including 1 patient that was weaned off completely. The number of OCS-requiring exacerbations reduced from a median of 2.5 (1–4.5) at baseline to 1 (0–2) at follow-up (*P* < 0.001). The need for SABA rescue therapy also decreased from a median of 10 (3–16) puffs/day to 2 (0.4–10) puffs/day (*P* = 0.001).

No significant differences in any of the measured lung function parameters were seen after BT (Table 2).

### Ventilation heterogeneity

**Fig. 1:**
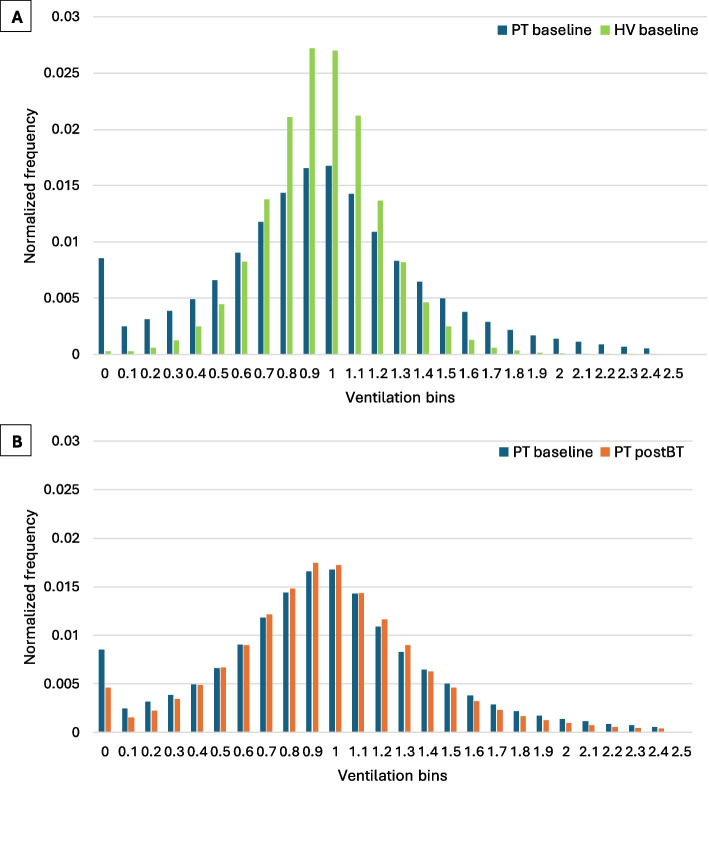
Pooled normalized ventilation histogram between patients and healthy volunteers at baseline (**A**), and between patients before and after bronchial thermoplasty (**B**). Patients show a more heterogeneous distribution of ventilation compared to healthy volunteers. Post bronchial thermoplasty, patients show a shift in the frequency distribution of ventilation from the lower and higher ends towards the center, corresponding to an improvement in ventilation heterogeneity. *BT, bronchial thermoplasty; HV, healthy volunteers; PT, patients*

At baseline, patients with asthma had a significantly higher RVent VDP (20.0 ± 14.5 vs 3.8 ± 2.2%, *P* < 0.001), FVL-CM VDP (23.7 ± 17.8 vs 2.4 ± 2.3%, *P* < 0.001), and IQD (0.61 ± 0.27 vs 0.32 ± 0.05, *P* < 0.001) than healthy volunteers. Differences in their pooled normalized histograms were also visible – wider and flatter in patients (more heterogeneous ventilation), compared with narrower and taller in healthy volunteers (more homogeneous ventilation) (Table 2 and Fig. 1a).

Post BT, ventilation heterogeneity was improved in patients with asthma, as evidenced by the significant reductions in RVent VDP (20.0 ± 14.5 vs 15.5 ± 11.7%, *P* = 0.04), FVL-CM VDP (23.7 ± 17.8 vs 18.7 ± 13.9%, *P* = 0.04), and IQD (0.61 ± 0.27 vs 0.53 ± 0.22, *P* = 0.04) at 6-months follow-up compared to baseline. Reduced ventilation heterogeneity was evident by the shift in the frequency distribution of the pooled normalized histogram, from the lower and higher ends towards the centre (Table 2 and Fig. 1b).

**Fig. 2:**
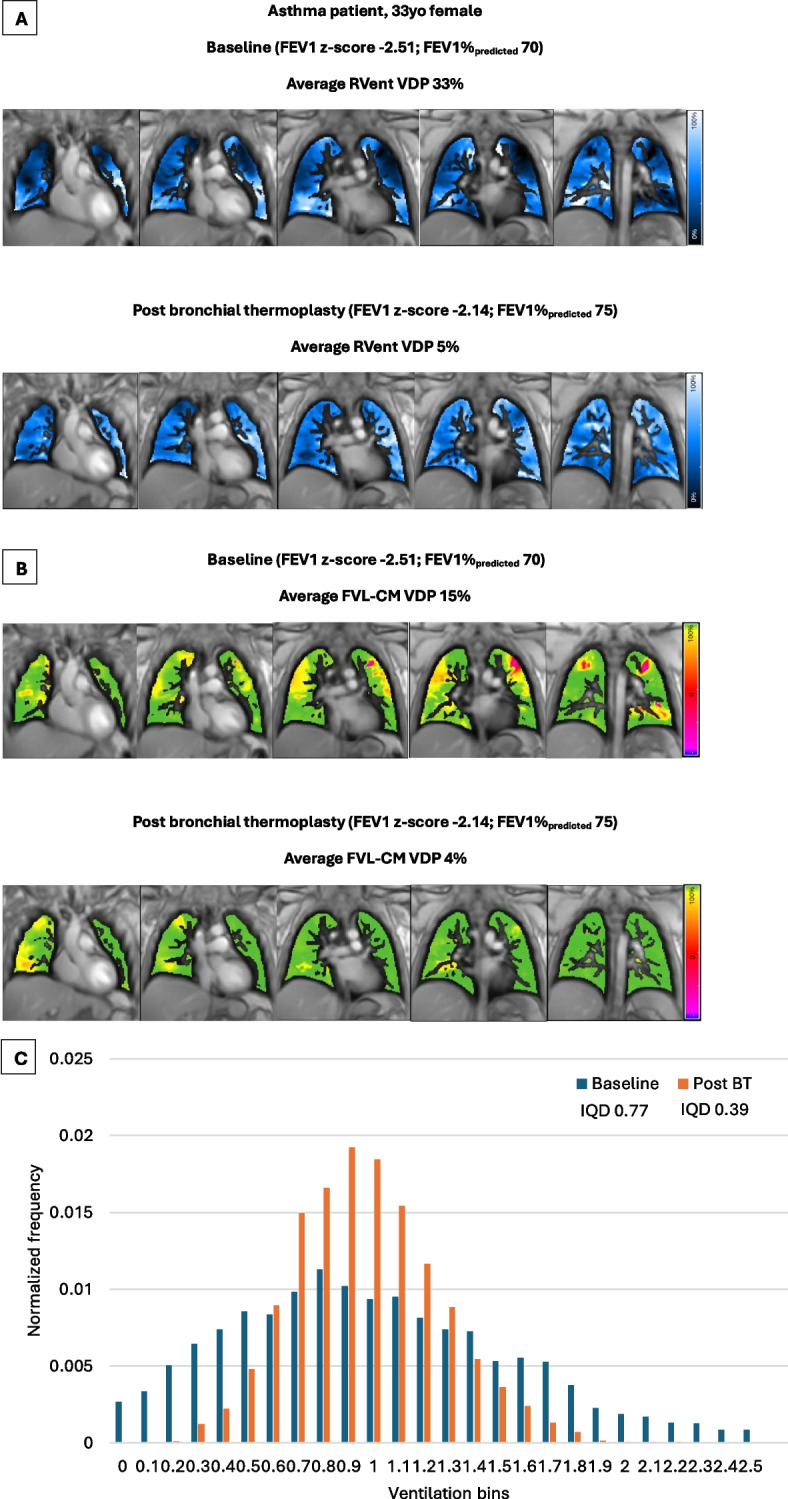
PREFUL regional ventilation (**A**) and flow volume loop cross-correlation maps (**B**) before and after bronchial thermoplasty in a 33 year old female with severe asthma. The images show a high ventilation defect burden at baseline, which improves after bronchial thermoplasty. Normalized ventilation histogram (**C**) of the same patient showing a reduction in ventilation heterogeneity post bronchial thermoplasty represented by a shift in the frequency distribution of ventilation from the lower and higher ends towards the center. *BT, bronchial thermoplasty; FEV1, forced expiratory volume in 1 s; FVL-CM, flow volume loop cross-correlation map; IQD, interquartile distance; RVent, regional ventilation; VDP, ventilation defect percentage*

Figure 2 shows the reduction in ventilation heterogeneity for a representative patient with asthma.

### Responder analysis

Seventeen out of 21 patients (81%) responded to BT (ACQ improved by > 0.5). The 4 non-responders were compared with their counterparts across a range of demographic, clinical, PREFUL MRI and lung function variables (Table 3). Responders were found to have a significantly higher baseline FEV1 z-score (−1.56 ± 1.41 vs −3.59 ± 1.50, *P* = 0.02; FEV1%_pred_ 76.0 ± 22.6 vs 47.5 ± 22.4, *P* = 0.04) and FER z-score (−1.56 ± 1.46 vs −3.61 ± 0.80, *P* = 0.02; FER% 67.9 ± 13.3 vs 49.3 ± 13.7%, *P* = 0.02), and a significantly lower baseline RVent VDP (16.7 ± 12.6 vs 34.3 ± 15.1%, *P* = 0.03), and IQD (0.55 ± 0.24 vs 0.88 ± 0.25, *P* = 0.02) compared to non-responders. These between group differences persisted post BT.

**Table 3:** Difference between BT responders and non-responders

Variable	Baseline			6-months		
	Responder (*N* = 17)	Non-responder (*N* = 4)	*P* Value	Responder (*N* = 17)	Non-responder (*N* = 4)	*P* Value
Age (years)	51.1 ± 17.7	49.5 ± 20.8	0.88	-	-	-
Males/Females	14 (82.4)/3 (17.6)	3 (75)/1 (25)	0.60	-	-	-
BMI (kg/m^2^)	33.1 ± 6.7	26.6 ± 7.5	0.10	-	-	-
Number of BT activations	311.3 ± 81.9	242.0 ± 72.4	0.14	-	-	-
Change in ACQ post BT	-	-	-	−1.69 ± 0.82	−0.15 ± 0.25	< 0.001
RVent VDP (%)	16.7 ± 12.6	34.3 ± 15.1	0.03	11.8 ± 8.6	31.3 ± 10.4	< 0.001
FVL-CM VDP (%)	22.6 ± 19.4	28.5 ± 8.8	0.57	16.6 ± 14.5	27.8 ± 6.2	0.15
IQD	0.55 ± 0.24	0.88 ± 0.25	0.02	0.45 ± 0.13	0.87 ± 0.23	< 0.001
Pre-bronchodilator FEV1 z-score/ %_pred_	−1.56 ± 1.41/ 76.0 ± 22.6	−3.59 ± 1.50/ 47.5 ± 22.4	0.02/ 0.04	−1.55 ± 1.05/ 77.7 ± 17.0	−3.89 ± 1.17/ 43.5 ± 18.8	< 0.001/ 0.002
Pre-bronchodilator FER z-score/ FER%	−1.56 ± 1.46/ 67.9 ± 13.3	−3.61 ± 0.80/ 49.3 ± 13.7	0.02/0.02	−1.34 ± 1.12/ 68.8 ± 11.3/	−3.85 ± 0.97/ 45.3 ± 16.3	< 0.001/ 0.003

### Correlations

**Fig. 3:**
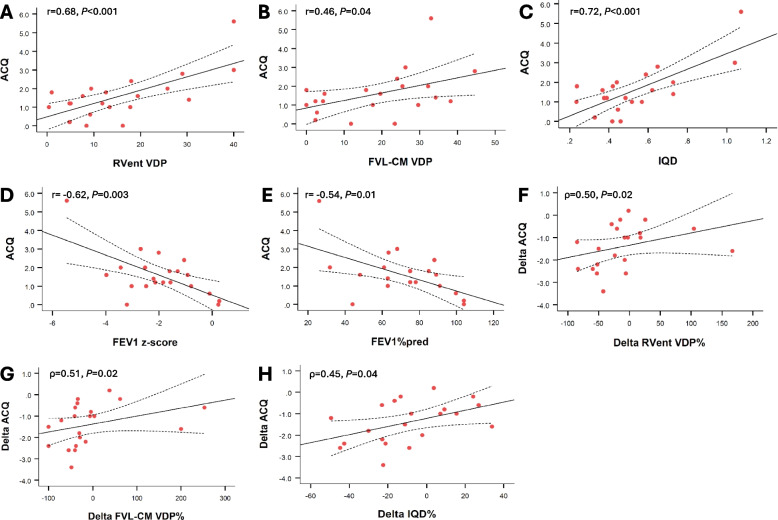
Correlations between post BT ACQ and post BT PREFUL MRI and lung function variables (**A**-**E**) – RVent VDP (r = 0.68, *P* < 0.001) (A), FVL-CM VDP (r = 0.46, *P* = 0.04) (**B**), IQD (r = 0.72, *P* < 0.001) (**C**), pre-bronchodilator FEV1 z-score (r = −0.62, *P* = 0.003) (**D**), and pre-bronchodilator FEV1_%pred_ (r = −0.54, *P* = 0.01) (**E**) – and between delta ACQ and delta PREFUL MRI variables (**F**–**H**) – delta RVent VDP% (ρ = 0.50, *P* = 0.02) (**F**), delta FVL-CM VDP% (ρ = 0.51, *P* = 0.02) (**G**), and delta IQD% (ρ = 0.45, *P* = 0.04) (**H**). Delta ACQ corresponds to the change in ACQ post BT. Delta RVentVDP%, FVL-CM VDP% and IQD% represent the percentage change in said variables post BT. The solid line represents the regression line and the dashed lines show the 95% confidence bands. *ACQ, asthma control questionnaire; FEV1, forced expiratory volume in 1 s; FVL-CM, flow volume loop cross-correlation matric; IQD, interquartile distance; RVent, regional ventilation; VDP, ventilation defect percentage*

The relationships between ACQ and various PREFUL MRI and lung function variables were also explored. Significant correlations were found between post BT ACQ and post BT RVent VDP (r = 0.68, *P* < 0.001), FVL-CM VDP (r = 0.46, *P* = 0.04), IQD (r = 0.72, *P* < 0.001), and pre-bronchodilator FEV1 z-score (r = −0.62, *P* = 0.003; FEV1%_pred_ r = −0.54, *P* = 0.01). Significant correlations were also observed between ΔACQ and ΔRVent VDP (ρ = 0.50, *P* = 0.02), ΔFVL-CM VDP (ρ = 0.51, *P* = 0.02), and ΔIQD (ρ = 0.45, *P* = 0.04), where Δ represents the change between post BT and baseline. All correlations remained significant after adjusting for multiple comparisons using the Hochberg method (Fig. 3).

## Discussion

This is the first study to assess changes in ventilation heterogeneity using PREFUL MRI in patients undergoing BT. The most important finding of this study is the demonstration that clinical improvements after BT were accompanied by significant reductions in ventilation heterogeneity without detectable changes in lung function. These findings are consistent with mathematical modelling of the physiological behaviour of the asthmatic lung when subjected to BT [13], and support our hypothesis that BT reduces ventilation heterogeneity. These results also provide further support of the complementary role of PREFUL MRI in assessing therapeutic response in asthma and other lung diseases.

Patients recruited in this study were severely affected by their asthma, as evidenced by persistent airflow obstruction on lung function, high symptom burden, and frequent exacerbations despite being on GINA step 4–5 treatment. This included a third of patients receiving treatment with an asthma biologic (a further 28.5% discontinued biologics prior to BT due to lack of efficacy) and just under 25% requiring maintenance OCS. It therefore follows that patients, with underlying pathologies of inflammation, mucus and high smooth muscle tone, should have worse ventilation heterogeneity when compared to healthy volunteers. Indeed, this was observed in the current study where patients not only exhibited a significantly higher VDP (RVent and FVL-CM) and IQD when compared to healthy volunteers, but also showed a strikingly different pooled normalized histogram (Fig. 1a).

Following BT, significant and substantive improvements in ACQ, maintenance OCS dose, OCS-requiring exacerbations and SABA usage were reported along with significant reductions in ventilation heterogeneity. In particular, VDP (RVent and FVL-CM) and IQD were noted to reduce by ~ 22% and ~ 13% respectively in patients post BT. Furthermore, a shift in the frequency distribution of the pooled normalized histogram from the lower and higher ends towards the centre was also seen, corresponding to a more homogeneous ventilation post BT. These findings, together with previous research demonstrating an increase in airway volume (and reduction in ASM) post BT [2, 14, 15], provide mounting cumulative evidence that treatment of large airways with BT facilitates more advantageous flow profiles in the lung periphery i.e. structural changes in the treated large airways can induce downstream functional changes in the untreated smaller airways [12, 13].

Despite the aforementioned improvements in clinical outcomes and ventilation heterogeneity, no significant change was noted in any of the measured lung function parameters after BT. While it is generally accepted that the effects of BT are not captured by spirometry or oscillometry [1, 6, 9, 11, 47], this likely reflects a failure of these traditional functional metrics, including FEV1, which is currently regarded as the clinical gold standard, in detecting physiological changes underpinning improved asthma control, such as those induced by BT and potentially other therapies. This has important implications in the design of future clinical trials and highlights the value of functional lung imaging, of which PREFUL MRI is one modality, as a complementary tool in assessing therapeutic response in lung disease.

Significant correlations were found between post BT ACQ and post BT VDP (RVent and FVL-CM), IQD, and FEV1 z-score/%_pred_, suggesting that patients who were the most symptomatic post BT (i.e. non-responders) also had the worse ventilation heterogeneity and poorer lung function. Significant correlations were also seen between ΔACQ and ΔRVent VDP, ΔFVL-CM VDP, and ΔIQD, implying that patients who had the biggest response to BT, also had the largest reduction in markers of ventilation heterogeneity. These findings are further supported by the group comparison showing that BT responders, defined as those with a greater than 0.5 unit improvement in ACQ post BT, exhibited significantly less ventilation heterogeneity (RVent VDP and IQD) and better lung function (higher FEV1 and FER) both at baseline and post BT when compared to non-responders.

The VDP (RVent and FVL-CM) results reported here are largely consistent with that published in the literature [24, 29, 32, 34, 48, 49]. In addition to VDP, we also utilized IQD as a measure of ventilation heterogeneity. IQD has previously been reported to be sensitive to changes induced by bronchodilator therapy and BT in patients with asthma [12, 24]. Unlike VDP, which focuses on “defects” based on values below a pre-defined threshold, IQD incorporates the entire spectrum of ventilation, and may provide different insights into the underlying ventilation compared to VDP alone.

Since its inception in 2010, the uptake of BT has been low [50] despite a randomized placebo-controlled trial and several large longitudinal studies showing sustained clinical benefits up to 10 years post treatment [1, 4–6]. Although the availability of asthma biologics has partially contributed to this, we believe a major reason for clinician reluctance to prescribe BT is the lack of a clear understanding of its mechanism of action, with many believing its effects to be placebo driven. The current study adds to the growing body of evidence [2, 12, 14, 15], and provides further proof that BT does indeed induce non-placebo physiological changes, and that this leads to improvements in patient outcomes which are not readily captured using traditional lung function tests.

In 2022, BSC announced the discontinuation of sales of the Alair Bronchial Thermoplasty System globally, citing an unfavourable commercial environment. While catheters continued to be available through December 31 st 2024, this announcement marked the beginning of the end of the BT era [51], with direct consequences for the large cohort of asthma patients who do not qualify for, or fail to achieve optimal disease control with biologics. Although it remains possible that an alternate system will emerge in future (clinicaltrials.gov identifier: NCT03765307; Registered 2018/12/5), ongoing research is crucial to identifying other therapies to address this unmet clinical need. With the reduced availability of BT going forward, the findings of the present study are novel and provide important information on the physiological changes expected with future therapies that seek to reduced ASM mass [52–54].

There are several limitations that need to be acknowledged. Firstly, interpretation of the results is limited by the small sample size and lack of a placebo-controlled group. The detection of significant differences despite the small sample size represents a strength of this study, which also includes a well characterized patient group. Secondly, though validated, the use of symptom scores is inherently associated with biases arising from patient factors e.g. recall bias. Thirdly, PREFUL MRI provides only an indirect assessment of ventilation, with the potential to be less sensitive than other imaging modalities, such as hyperpolarized gas imaging, that are able to directly probe lung ventilation. However, the greater accessibility of PREFUL makes it an attractive alternative to tracer gas methodologies and findings to date suggest outcomes are sensitive to therapeutic response [24–27, 31, 33, 38].

## Conclusion

Clinical benefits after BT are accompanied by significant reductions in ventilation heterogeneity that are undetected using traditional lung function test. These findings are consistent with current modelling, advances our understanding of the mechanisms of action of BT, and provide insights as to how we, as a community, missed BT’s effect in its early days. Beyond BT, these findings underscore the complementary role of functional lung imaging in the study of other pulmonary diseases for which traditional lung function test may be insensitive at detecting a therapeutic response.

## Data Availability

The datasets used and/or analysed during the current study are available from the corresponding author on reasonable request.

## Abbreviations

ACQ: Asthma control questionnaire
ASM: Airway smooth muscle
BT: Bronchial thermoplasty
DLCO: Diffusing capacity of the lung for carbon dioxide
ERS/ATS: European Respiratory Society/American Thoracic Society
FER: Forced expiratory ratio
FEV1: Forced expiratory volume in 1 s
FVL CM: Dynamic flow volume loop cross-correlation metric
GINA: Global Initiative for Asthma
IQD: Interquartile distance of ventilation distribution
MRI: Magnetic resonance imaging
OCS: Oral corticosteroids
PREFUL: Phase-resolved functional lung
RVent: Static regional ventilation
SABA: Short acting beta-2 agonist
VDP: Ventilation defect percentage
